# Longevity Factor FOXO3: A Key Regulator in Aging-Related Vascular Diseases

**DOI:** 10.3389/fcvm.2021.778674

**Published:** 2021-12-23

**Authors:** Yan Zhao, You-Shuo Liu

**Affiliations:** ^1^Department of Geriatrics, The Second Xiangya Hospital, Central South University, Changsha, China; ^2^Institute of Aging and Age-Related Disease Research, Central South University, Changsha, China

**Keywords:** FOXO3, aging, vascular aging, vascular homeostasis, cardiovascular disease

## Abstract

Forkhead box O3 (FOXO3) has been proposed as a homeostasis regulator, capable of integrating multiple upstream signaling pathways that are sensitive to environmental changes and counteracting their adverse effects due to external changes, such as oxidative stress, metabolic stress and growth factor deprivation. FOXO3 polymorphisms are associated with extreme human longevity. Intriguingly, longevity-associated single nucleotide polymorphisms (SNPs) in human *FOXO3* correlate with lower-than-average morbidity from cardiovascular diseases in long-lived people. Emerging evidence indicates that FOXO3 plays a critical role in vascular aging. FOXO3 inactivation is implicated in several aging-related vascular diseases. In experimental studies, FOXO3-engineered human ESC-derived vascular cells improve vascular homeostasis and delay vascular aging. The purpose of this review is to explore how FOXO3 regulates vascular aging and its crucial role in aging-related vascular diseases.

## Background

Cardiovascular disease (CVD) is the leading cause of morbidity and mortality in individuals aged 65 years and above (1). Vascular aging has been implicated as a driver of a number of aging-related vascular disorders (2). A large clinical study identified two specific age-related arterial phenotypes, endothelial dysfunction, and increased arterial stiffness as independent predictors for future diagnosis of CVD (3). At the macro level, aging vessels exhibit luminal expansion, diffused stiffness, wall thickening, and blunted angiogenesis (4, 5). Microscopically, aging vessels undergo vascular cell senescence and loss of vascular homeostasis, resulting in inflammation, oxidative stress, and calcification of blood vessels (4). The pace of vascular aging differs in individuals due to differences in their genetics and environment background.

The insulin/IFG-1 signaling (IIS) pathway is one of the major pathways involved in the regulation of aging rate, which negatively influences the activity of forkhead box O3 (FOXO3) (6). The first identified FOXO member, DAF-16/FOXO3, has been shown to prolong lifespan in *C. elegans* by regulating insulin-like metabolic signaling (7, 8). Additionally, studies have demonstrated that the IIS pathway is associated with an extended lifespan in a variety of species including worms, yeasts, flies, and mice (9). To assess the genetic contributions of genes associated with IIS signaling to human longevity, researchers performed a nested case-control study on 5 prospective longevity genes and found that *FOXO3* variation was strongly correlated with human longevity (10). Subsequently, this finding was quickly duplicated in a variety of populations around the world (11–13). Five *FOXO3* single nucleotide polymorphisms (SNPs) were shown to have a significant correlation with longevity in a meta-analysis of 11 independent studies (14). *FOXO3* has been identified as the second most replicated gene associated with extreme human longevity (15). While *FOXO3* is a convincing longevity gene, the mechanism by which FOXO3 determines longevity remains unknown. Interestingly, long-lived individuals demonstrated some phenotypes associated with healthy aging, including a lower prevalence of CVD and cancer (10). Additionally, the longevity-associated *FOXO3* SNPs correlate with lower-than-average CVD morbidity in long-lived individuals (10, 16). Another study found that longevity-associated *FOXO3* genetic variants prolong lifespan only in individuals with cardiometabolic disease (CMD), but not in all individuals (17). These findings show that FOXO3 may maintain cardiovascular homeostasis, hence promoting longevity. A single-cell transcriptomic analysis of coronary arteries and aortas of young and elderly cynomolgus monkeys found that FOXO3 expression was downregulated in six subtypes of vascular cells in older monkeys compared to young monkeys (18). Although the underlying mechanisms are unknown, FOXO3 is require for maintaining vascular homeostasis under stressful conditions and preventing vascular aging. In this review, we will summarize the most recent findings on FOXO3 functions and mainly focus on its role in aging-related vascular diseases.

## Overview of FOXO3

FOXO proteins are ubiquitously expressed transcription factors that activate gene transcription when they recognize promoters containing the sequence 5′-TTGTTTAC-3′ (19). By integrating multiple upstream signaling pathways, FOXOs help maintain tissue homeostasis and counteract adverse effects of environmental changes such as oxidative stress, metabolic stress, and growth factor deprivation (20). The transcriptional targets of FOXOs include genes involved in cell cycle arrest (21), oxidative resistance (22), apoptosis (23), autophagy (24), DNA damage repair (25), and energy metabolism (26). The biological role of FOXOs is primarily to respond to stress conditions, rather than as an essential agent of normal physiology. In humans, the FOXO family comprises FOXO1, FOXO3, FOXO4, and FOXO6. FOXO3 has been associated with a number of age-related diseases, including cancer (27), CVD (28), intervertebral disc (IVD) degeneration (29), and neurodegenerative diseases (30). Particularly, the role of FOXO3 in CVD appears attractive.

## Regulation of FOXO3

Numerous upstream factors regulate FOXO3 via post-transcriptional or post-translational modifications. The exquisite regulatory network formed by diverse upstream regulators and downstream effectors of FOXO3 contributes to its responsiveness to environmental changes and plays an important role in maintaining homeostasis (20).

### MicroRNAs Contribute to Post-transcriptional Regulation of FOXO3

MicroRNAs (miRNAs) act as post-transcriptional regulators of gene expression (31). Numerous miRNAs, including miR-155, miR-132, miR-212, miR-223, miR-27a, miR-96, miR-30d, miR-182, miR-592, miR-1307 and miR-29a, bind to FOXO3 3′-UTR and inhibit its expression (27). Other miRNAs have an indirect effect on FOXO3, for example, by targeting upstream factors of FOXO3 to modulate its activity (32). Long non-coding RNAs (lncRNAs) and circular RNAs (circRNAs) are also known to regulate FOXO3 (33, 34). A comprehensive exploration of the relationship between non-coding RNAs and FOXO3 will help in the development of more effective chemotherapy.

### Post-translational Modifications of FOXO3

FOXO3 activity is mainly regulated by post-translational modifications (PTMs), including phosphorylation, acetylation, methylation, ubiquitination, glycosylation, prenylation, and sulphation. Most of these PTMs change the subcellular localization of FOXO3 and its DNA binding affinity (27). The subcellular localization of FOXO3 is essential for its activity and function (35).

#### Phosphorylation and Dephosphorylation

The primary regulator of FOXO3 activity is its translocation between the nucleus and cytoplasm. Phosphorylation is a critical PTM that regulates FOXO3 activity. Numerous kinases recognize specific sites on FOXO3 and may exert opposing effects on its activity (36). FOXO3 is inactive under normal conditions, due to negative regulation by IIS-PI3K-Akt signaling. Akt phosphorylates FOXO3 at three highly conserved residues, T32, S253, and S315, establishing docking sites for the chaperone 14-3-3, preventing FOXO3 from re-entering the nucleus (37). PTEN antagonizes the effect of PI3K and induces FOXO3 activation. When cells are stressed, such as when reactive oxygen species (ROS) levels are elevated, FOXO3 translocates into the nucleus and exhibits increased transcriptional activity (20).

The majority of phosphorylases, including extracellular signal-regulated kinase (ERK), IκB kinase isoform β (IKKβ), serum-and glucocorticoid-inducible kinases (SGK), and inhibitor of nuclear factor kappa-B kinase subunit epsilon (IKBKE) suppress FOXO3 activity (38). In comparison, FOXO3 is activated upon phosphorylation by c-Jun N-terminal kinase (JNK), mammalian sterile 20-like kinase 1 (MST1), and AMP-activated protein kinase (AMPK) (39–41). AMPK-mediated phosphorylation impacts FOXO3's interaction with cofactors but does not affect does not affect its subcellular localization (42). JNK inhibits insulin signal transduction on multiple levels by reducing the activity of insulin receptor substrate (IRS) and inducing the release of FOXO3 from 14-3-3, hence surpassing growth factor-induced FOXO3 inhibition (19).

#### Acetylation and Deacetylation

Nuclear FOXO3 is acetylated by p300 and CBP and deacetylated by deacetylases such as SIRT1 and SIRT2. The effect on acetylation and deacetylation on FOXO3's affinity for DNA is controversial (43, 44). Notably, the effects of SIRT1 on FOXO3 activity are not fixed rigidly, for instance, SIRT1 promotes the expression of target genes associated with antioxidant stress but suppresses the expression of proapoptotic genes (45).

Ubiquitination and methylation also act as regulators of FOXO3, with multiubiquitination resulting in FOXO3 degradation. Different PTMs may occur on the same lysine residues on FOXO3, for example, lysine residues deacetylated by SIRT1 might be ubiquitinated, thereby degrading FOXO3 (46). Alterations in PTMs associated with aging may contribute to the onset of some age-associated diseases.

## Role of FOXO3 in Vascular Aging

**Figure 1:**
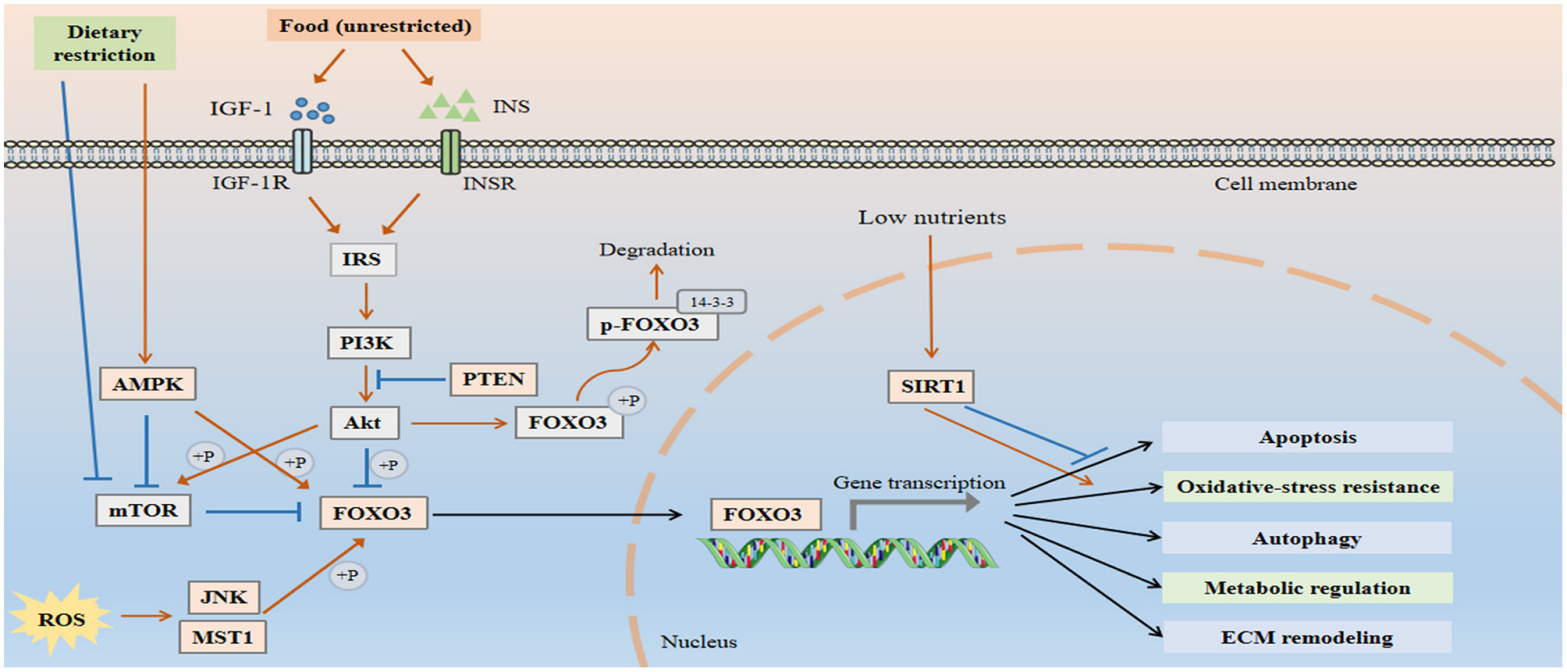
FOXO3 is an integrator of multiple signaling pathways to maintain vascular homeostasis. Under normal conditions, FOXO3 is inactive due to the negative regulation by IIS-PI3K-Akt pathway. Akt phosphorylates FOXO3 at three highly conserved residues T32, S253, and S315, thereby establishing docking sites for the chaperone protein 14-3-3 and preventing it from re-entering the nucleus. PTEN antagonizes the effect of PI3K and induces FOXO3 activation. When cells are exposed to stress, including growth factor deprivation, metabolic stress, and oxidative stress, FOXO3 translocates into the nucleus and exhibits increased transcriptional activity. FOXO3 regulates a number of cellular processes, including apoptosis, autophagy, oxidative resistance, and metabolism, all of which are involved in the pathological process of vascular aging.

Aging-associated mechanisms, including deregulated nutrient sensing, oxidative stress, and epigenetic changes in the vascular system may contribute to the pathogenesis of vascular aging. FOXO3 acts as an integrator of multiple signaling pathways involved in the maintenance of vascular homeostasis, and its dysregulation is implicated in a variety of vascular disorders (Figure 1).

### FOXO3 and Oxidative Stress

Oxidative stress is a major driving force for vascular aging. Age-related increases in reactive oxygen species (ROS) result in endothelial dysfunction and arterial stiffness (47, 48). Endothelium-derived nitric oxide (NO) possesses anti-inflammatory, anti-thrombotic, and anti-leukocyte adhesion properties. Under pathological condition, ROS inactivates NO, which may contribute to the development of atherosclerosis (49). Exercise can restore endothelium-dependent dilation in aged mice by increasing NO bioavailability and reducing oxidative stress (50).

FOXO3 deletion results in an increase in ROS in mouse hematopoietic stem cells (51). FOXO3 is indispensable for the antioxidant-mediated protection in cardiovascular system. FOXO3 protects vascular endothelial cells (ECs) and vascular smooth muscle cells (VSMCs) against oxidative stress injury by up-regulating the expression of antioxidant enzymes such as catalase and manganese-superoxide dismutase (MnSOD) (52, 53).

### FOXO3 and Dysregulated Nutrient-Sensing Pathways

#### AMPK and mTOR

AMPK and mTOR are key regulators of energy homeostasis. AMPK promotes ATP synthesis in response to an energy deficit caused by glucose deprivation or exercise (54). Activated AMPK regulates cell growth and metabolism at low energy levels by phosphorylating a range of substrates (55). In VECs, AMPK activates endothelial nitric oxide synthase (eNOS), phosphorylating it directly and so promoting NO production (56). Second, AMPK activation inhibits the generation of inflammatory chemokines in VECs by attenuating nuclear factor-kappaB (NF-κB) signal transduction (54). Two more studies demonstrate that AMPK signaling in ECs is required for angiogenesis in response to hypoxic stress and differentiation of endothelial progenitor cells (57, 58). However, AMPK activity is reduced in the aorta and cerebral arteries of old rodents (50, 59).

mTOR is a key regulator of anabolic processes and its activity decreases in response to nutrient deprivation. Decreased mTOR activity influences aging and longevity in invertebrates and mice (60). Numerous studies have demonstrated that inhibiting mTOR delays EC senescence (61, 62). Additionally, mTOR inhibition mediates the phenotypic transition of VSMCs by blocking the PDGF-induced contractile VSMC reduction (63). Rapamycin, an mTOR inhibitor, suppresses oxidative stress and vascular stiffness, revering the effects of age-related arterial dysfunction (64).

Between AMPK and mTOR, there are intricate and precise regulatory mechanisms that efficiently regulate energy metabolism. Akt, a positive regulator of energy metabolism, inhibits AMPK and promotes mTOR complex 1 (mTORC1) activation (65). AMPK is activated in response to energy stress, whereas mTORC1 is inactivated (66). Activated AMPK phosphorylates FOXO3 (42), which is an effector of AMPK-mediated apoptosis (67) and hypoxia-induced autophagy (68). Additionally, FOXO3 may suppress mTORC1 activity by inhibiting the regulatory associated protein of mTOR (Raptor) (69). AMPK promotes FOXO3 activation and inhibits mTOR, which seems to be protective in response to hypoxia, ROS, and starvation (70).

#### SIRTs

SIRTs are also activated in cells with inadequate nutritional status to increase their resistance to stress. The activated SIRT family members have anti-inflammatory, anti-oxidative stress and anti-senescence effects in the vasculature (71–74). SIRT1 acts as an anti-atherosclerotic factor in mice by preventing DNA damage (75). However, endogenous SIRT1 expression decreases with age (76). Decreased SIRT1 levels also contribute to vascular endothelial dysfunction associated with aging through its modulation of eNOS acetylation (77). Similarly, SIRT6 protects against DNA damage, telomere dysfunction, senescence, and atherosclerosis in vascular cells (78, 79).

Chronic hyperglycemia induces accelerated vascular aging. SIRT1-mediated deacetylation of FOXO3 is important for VECs survival under high-glucose conditions (80, 81). High glucose levels suppress the expression of SIRT1 and FOXO3 in VECs. SIRT1 overexpression protects VECs from high glucose-induced apoptosis (81). Additionally, the AMPK/SIRT1/FOXO3 signaling pathway affects the phenotypic switching of VSMCs (82). Moreover, the role of SIRT1 in ameliorating oxidative stress is associated with FOXO3 activation (22). SIRT1 enhances catalase activity and induces MnSOD expression by deacetylating FOXO3 to control intracellular ROS levels (83).

Although caloric restriction (CR) slows the aging process and decreases diabetes and CVD mortality, the underlying mechanisms are unknown (84). Numerous studies have highlighted the importance of nutrient-sensitive protein pathways such as AMPK, mTOR, SIRTs, and the insulin pathway (41). FOXO3 mediates cellular response to CR. By serving as a downstream effector for the insulin, AMPK and SIRTs pathways, FOXO3 stimulates the expression of stress genes in response to nutritional deficiency (85).

### FOXO3 and Apoptosis

Apoptosis is evident in ECs and VSMCs in atherosclerotic plaques (86–88). FOXO3 up-regulates the expression of numerous apoptosis-related genes, including FasL, Bim, Puma, TRAIL, and Noxa (89). Bim is a proapoptotic member of the Bcl-2 family, and its expression is suppressed by Akt activation in VSMCs expressing wild-type FOXO3, but not in FOXO3 mutant cells (90). Apoptosis is an essential process by which unwanted or abnormal cells are removed during development. Apoptosis, however, may result in microvascular rarefaction and aneurysm in the vasculature. VSMCs apoptosis may even cause atherosclerotic plaque instability and rupture (91).

### FOXO3 and Autophagy

Autophagy maintains homeostasis by removing damaged organelles and misfolded proteins (92). Autophagy has been shown to decrease with aging in animal models (93). Overexpression of autophagy-related gene 5 (*ATG5*) induces autophagy and prolongs life span in mice (94). In the vascular system, autophagy is associated with diverse physiological and pathophysiological processes, including angiogenesis, vascular calcification, and atherosclerosis (95). Reduced autophagy markers in senescent ECs may impair arterial endothelium-dependent dilatation (96). Autophagy is also reported to preserve arterial endothelial function by increasing NO bioavailability and reducing inflammation and oxidative stress (96). Additionally, spermidine-induced autophagy improves NO bioavailability and reduces arterial stiffness in aged mice (97).

Numerous autophagy-related genes, including *ATG12, BNIP3, ATG8*, and *GABARAPL1* are targets of FOXO3 (24, 98). FOXO3 is an important pro-autophagic factor in cardiomyocytes (99). In cardiac microvascular endothelial cells (CMECs), hypoxia suppresses phosphorylation of FOXO3 which induces autophagy formation (100). FOXO3 accumulation and nuclear translocation also elevate ATG protein levels in renal tubular epithelial cells, thus complementing the core component of autophagy (101).

### FOXO3 and Epigenetics

Aging is a complex process driven by genetic and environmental factors. Epigenetics, an important interface between genetic and environmental factors influences aging as well as the occurrence and progression of aging-related disorders (102). Epigenetics, including DNA methylation patterns, histone modifications, and non-coding RNA regulation, play a crucial role in vascular aging (103).

MiRNA expression in VECs and VSMCs may correlate with vascular aging (104). Various miRNAs that directly influence FOXO3 expression, including miR-27a, miR-155, miR-233, and miR-29a affect vascular pathological processes. MiR-155 inhibits EC proliferation and migration, which eventually disrupts endothelial barriers (105). MiR-148a-3p upregulation in atherosclerotic patients suppresses FOXO3 expression and impairs EC proliferation and migration, ultimately aggravating atherosclerosis (106). Upregulation of a disintegrin and metalloproteinase with thrombospondin motifs-7 (ADAMTS-7) by miR-29a repression attenuates VSMC calcification (107). MiR-34a, upregulated in atherosclerotic plaques (108), targets SIRT1 3′ UTR to suppress SIRT1 expression (109). The role of SIRT1 in reducing oxidative stress depends on FOXO3 activation. Notably, the reversibility of epigenetic changes is a promising approach for developing epigenome-influencing interventions against cardiovascular disorders.

### FOXO3 and ECM Remodeling

Aging alters extracellular matrix (ECM) synthesis and cell-ECM interactions (110). Decreased elastin synthesis with age reduces the elasticity and resilience of the vascular wall, impairing its ability to cope with mechanical damage and sudden changes in pulsatile pressure waves (111). Increased collagen synthesis in arterial walls linked with aging contributes to vascular fibrosis and arteriosclerosis (111). Aging also affects the secretion phenotype of VECs and VSMCs and affects matrix metalloproteinase (MMP) secretion (112). Elevated MMP activation by high ROS levels impairs the structural integrity of the vascular system and promotes pathological remodeling, contributing to the possibility of aneurysm formation and vascular rupture (112). Aging-related ECM remodeling also obstructs microvascular barrier function (113).

Studies on the effect of FOXO3 on MMPs have yielded inconsistent results. MMP13, MMP2 and MMP3 are considered FOXO3 targets. Activated FOXO3 induced ECM breakdown via MMP13 activation (28). Apelin, an adipocytokine, induces VSMC migration which is a critical event in atherosclerosis progression. Apelin promotes Akt-mediated phosphorylation of FOXO3, which enhances FOXO3 translocation from the nucleus to the cytoplasm and increases MMP2 levels (114). Additionally, FOXO3 phosphorylation has been shown to inhibit MMP3 promoter activity (115). ECMs are important in EC survival. Constitutively active FOXO3 enhances MMP3 expression and leads to EC apoptosis, which can be reversed by an MMP inhibitor, suggesting a novel mechanism of FOXO3-mediated EC apoptosis (116).

## FOXO3 in Aging-Related Vascular Diseases

**Figure 2:**
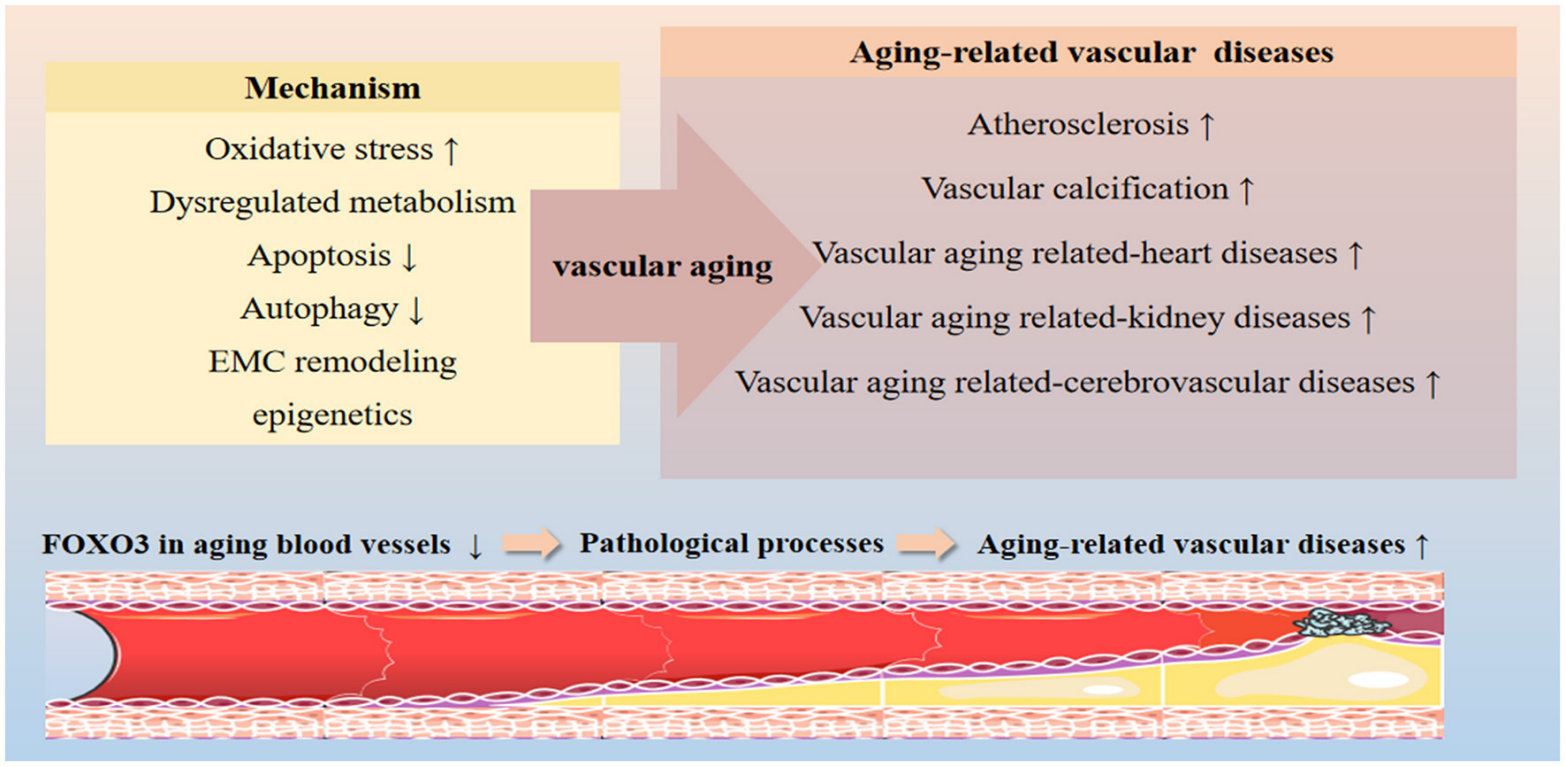
Effects of FOXO3 on vascular aging-related diseases. FOXO3 participates in various cellular processes implicated in the progression of vascular aging, including oxidative resistance, apoptosis, autophagy, energy metabolism, and ECM remodeling processes by targeting the expression of effector genes. FOXO3 is a key protective factor in maintaining vascular homeostasis. Dysregulation of FOXO3 has been shown to contribute to a variety of vascular aging-related diseases, including atherosclerosis, vascular calcification, hypertension, and vascular aging-related heart diseases, kidney diseases, and cerebrovascular diseases.

FOXO3 influences the progression of aging-related vascular diseases by regulating the expression of genes involved in oxidative stress, apoptosis, autophagy, and metabolic stress (Figure 2). In the following section, we will discuss the role of FOXO3 in diseases such as atherosclerosis, vascular calcification, hypertension, and vascular aging-related heart diseases, kidney diseases, and cerebrovascular diseases (Table 1).

**Table 1:** FOXO3 cellular signaling in vascular aging-related diseases.

**Diseases**	**Biological effects of FOXO3**	**References**
Atherosclerosis	The longevity-associated G allele of *FOXO3* SNP rs2802292 is protective against CAD mortality	(117)
	Phosphorylated FOXO3 is increased in human carotid atherosclerotic plaques	(114)
	FOXO3 phosphorylated by Akt protects VSMCs from oxidative stress-induced apoptosis	(90)
	FOXO3 phosphorylation promotes VSMC migration	(114)
	Up-regulated AMPK/SIRT1/FOXO3 signaling increases the protein levels of the VSMC contractile markers	(84)
	FOXO3 suppresses VSMC proliferation and neointimal hyperplasia by downregulating CYR6	(122)
	Decreased FOXO3 inhibits NLRP3-mediated EC pyroptosis	(123)
	Melatonin ameliorates atherosclerosis by regulating SIRT3/FOXO3/Parkin signaling	(124)
Vascular calcification	FOXO3 phosphorylated by Akt promotes VSMC calcification	(125)
Vascular aging related-heart diseases	FOXO3-null mice developed dilated cardiomyopathy within 12 weeks of age	(129)
	Reduced FOXO3 in senescent cardiac microvascular ECs leads to inhibition of proliferation and tube formation	(130)
	FOXO3 overexpression suppressed the aging of cardiac microvascular ECs by regulating the antioxidant/ROS/p27 (kip1) pathway	(130)
	Downstream proapoptotic genes mediated by FOXO3 are activated in aging cardiomyocytes	(133)
	FOXO3 deficiency in the heart enhances paraquat-induced aging phenotypes	(134)
	FOXO3 activation and expression are reduced in cardiac fibroblasts. Overexpression of FOXO3 inhibited while knockdown of FOXO3 enhanced TFGβ1-induced cardiac myofibroblasts transformation	(135)
Vascular aging related-kidney diseases	FOXO3 promotes the expression of Atg proteins to sustain autophagy in the chronically hypoxic kidney	(101)
	FOXO3 deacetylated by SIRT1 induces the expression of BNIP3, and ultimately promotes mitochondrial autophagy, and protect aging kidneys	(137)
	Tubular deletion of FOXO3 aggravates renal structural and functional damage	(138)
	Activated FOXO3 in mice with unilateral ureteral obstruction contributes to high levels of autophagy	(139)
	FOXO3 ameliorates oxidative stress, suppressing renal fibrosis induced by diabetes and hypertension	(141–143)
Vascular aging related-cerebrovascular diseases	Increased risks of stroke are observed for *FOXO3* block-A haplotype 2 “GAGC” and haplotype 4 “AAAT” carriers	(144)
	Overexpressed FOXO3 in the cerebral cortex of MCAO mice promotes neuronal death	(145, 146)
	AMPK/FOXO3 and PTEN-Akt-FOXO3 pathways regulate neuronal apoptosis after hypoxia-ischemia in the developing rat brain	(67, 147)
	Increased FOXO3 activation has a protective effect on cerebral ischemia-reperfusion injury by promoting autophagy	(148)
Primary hypertension	Longevity related *FOXO3* variants may contribute to low risk for essential hypertension in Japanese women	(150)

### The Role of FOXO3 in Atherosclerosis

FOXO3 genotypes are associated with the risk of death from coronary artery disease (CAD) in the elderly. The longevity-associated G allele of *FOXO3* SNP rs2802292 is protective against CAD mortality (117). The plasma TNF-α levels of G allele carriers were lower than that of non-carriers, implying that FOXO3-mediated inflammation inhibition is a protective mediator of CAD death risk (117). LDL-cholesterol is an important risk factor for CVD. FOXO3 and SIRT6 regulate LDL cholesterol homeostasis by regulating the *PCSK9* gene expression, which lowers LDL levels by inhibiting LDL receptor degradation (118).

Under normal growth conditions, FOXO3 is negatively regulated by IGF-1/PI3K/Akt signaling (20). Age-related decline of IGF-1R suppresses Akt/FOXO3 in VSMCs (119). Low levels of VSMC apoptosis occur in atherosclerotic plates. Compared with normal VSMCs, VSMCs in the atherosclerotic plate express lower IGF-1R levels and exhibit higher apoptosis. IGF-1R overexpression is reported to protect VSMCs from oxidative stress-induced apoptosis by up-regulating Akt-mediated phosphorylation of FOXO3 (90).

VSMC migration is a key event in the development of atherosclerosis. In human carotid atherosclerotic plaques, apelin induces FOXO3 phosphorylation in a dose-dependent manner, and mediates VSMC migration (114). Glucagon-like peptide-1 receptor (GLP-1R) is widely expressed in various cell types, such as VSMCs and cardiomyocytes (120). GLP-1R agonist exendin-4 not only inhibits angiotensin II-induced cell senescence but also inhibits PDGF-induced VSMC proliferation and migration (121). Exendin-4 has been shown to elevate the expression of VSMC contractile markers, Calponin and SM22α, by upregulating AMPK/SIRT1/FOXO3 signaling (82). Cysteine-rich angiogenic protein 61 (CYR61) has been implicated in restenosis after angioplasty. FOXO3, a CYR61 antagonist, inhibits VSMC proliferation and neointimal hyperplasia (122).

The inflammatory response mediated by the NOD-like receptor family pyrin domain-containing 3 (NLRP3) inflammasome is associated with atherosclerosis progression. MiR-30c-5p downregulates FOXO3 expression, inhibiting NLRP3-mediated EC pyroptosis in atherosclerosis (123). Melatonin has been demonstrated to ameliorate atherosclerosis by inhibiting NLRP3 inflammasome, which is regulated by SIRT3/FOXO3/Parkin signaling (124).

### The Role of FOXO3 in Vascular Calcification

Vascular calcification refers to the ectopic deposition of calcium salts in blood vessels. It is associated with vascular aging, atherosclerosis, advanced nephropathy, and diabetes (125). Runt-related transcription factor 2 (Runx2), a key osteogenic regulator, regulates VSMC osteogenic differentiation and vascular calcification in atherosclerosis (126). Akt activation is reported to contribute to oxidative stress-induced VSMC calcification by upregulating Runx2 expression (127). Deficiency in PTEN, an Akt inhibitor, results in sustained Akt activity, which results in FOXO3 phosphorylation, Runx2 ubiquitination, and VSMC calcification (125). Other members of the FOXO family have also been implicated in the regulation of vascular calcification. For example, FOXO1 dysregulation contributes to peripheral arterial calcification (128).

### The Role of FOXO3 in Vascular Aging Related-Heart Diseases

Left ventricular hypertrophy is a crucial feature of cardiac aging, leading to diastolic dysfunction, atrial fibrillation, and heart failure. Moreover, atherosclerotic diseases (e.g., coronary heart disease) might result in chronic myocardial insufficiency, ischemic heart disease, or even heart failure. FOXO3 plays important role in the maintenance of cardiac homeostasis. For example, FOXO3-null mice developed dilated cardiomyopathy within 12 weeks of age (129). Low expression of FOXO3 in senescent cardiac microvascular ECs suppressed the ability of cell proliferation and tube formation. Additionally, it has also been observed that FOXO3 overexpression slowed the senescence of cardiac microvascular ECs via modulating the antioxidant/ROS/p27 (kip1) pathway (130).

A previous study reported that compared with young people, persons above the age of 70 years had 30% fewer myocardial cells (131), which may be ascribed to apoptosis (132). The aged heart is more susceptible to ischemia-reperfusion injury. SIRT1 expression was significantly reduced in aged cardiomyocytes, while FOXO3-mediated antioxidant kinase decreased and apoptosis increased, aggravating myocardial ischemia-reperfusion injury (133).

Paraquat inhibits FOXO3 activation and induces cardiomyocyte senescence phenotype. FOXO3 silencing in the heart greatly accelerated the aging phenotypes induced by paraquat, including proliferation inhibition, apoptosis, and galactosidase activity. FOXO3 has also been shown to protect the heart against paraquat-induced aging phenotypes by upregulating the expression of antioxidant enzymes and inhibiting oxidative stress (134).

Cardiac fibroblasts (CFs) contribute to the maintenance of the ECM balance in the normal heart. Under normal conditions, CFs exist in a quiescent state and secrete only a small amount of ECM components. However, CFs differentiate into more active cardiac myofibroblasts (CMFs) under pathological conditions. This CMF conversion is a hallmark of cardiac fibrotic diseases such as heart failure and diabetic cardiomyopathy. TGF-β1 is a key protein that regulates CMF transformation. Previously, it was reported that TGF-β1 decreased FOXO3 expression in a concentration-dependent manner in CFs. Overexpression of FOXO3 inhibited whereas knockdown of FOXO3 enhanced TFGβ1-induced CMF transformation (135). Therefore, FOXO3 may act as a negative regulator of CMF conversion triggered by TGF-β1.

### The Role of FOXO3 in Vascular Aging Related-Kidney Diseases

Vascular aging increases the risk of chronic kidney disease (CKD). A previous investigation established that arterial stiffness contributed to the decline in kidney function (136). Hypoxia inhibits the hydroxylation of FOXO3 prolyl, thereby reducing the degradation of FOXO3. FOXO3 upregulates the expression of Atg proteins, which promote autophagy in chronically hypoxic kidneys (101). Calorie restriction maintains renal SIRT1 expression, and elevates BNIP3 expression by deacetylating FOXO3, which promotes mitochondrial autophagy and delays the effects of aging on kidneys (137). Previously, it was demonstrated that tubular deletion of FOXO3 aggravated renal structural and functional damage, leading to a more severe CKD phenotype (138). FOXO3 was discovered to be activated in mice with unilateral ureteral obstruction. Moreover, hypoxic proximal tubules activates autophagy in response to urinary tract obstruction (139).

Tissue fibrosis is a common manifestation of aging-related diseases. Currently, there are limited therapeutic targets to prevent fibrogenesis. As previously indicated, FOXO3 inhibits fibroblast activation and ameliorates fibrosis levels in many organs, including the kidney, liver, heart, and lung (140). Renal fibrosis, including glomerulosclerosis and tubulointerstitial fibrosis, is the pathological hallmark of CKD. FOXO3 ameliorates oxidative stress, thereby suppressing renal fibrosis associated with diabetes and hypertension (141, 142). FOXO3 was found to be directly regulated by miR-132 in a mouse model experiment. Indeed, silencing miR-132 delayed the progression of renal fibrosis, implying that miR-132 could be a potential therapeutic target for fibrosis treatment (143).

### The Role of FOXO3 in Vascular Aging Related-Cerebrovascular Diseases

During vascular aging, entry of high pulsating blood flow into small cerebral vessels may damage the cerebral microvessels, resulting in cerebrovascular diseases or cognitive impairment. Haplotype analyses of *FOXO3* revealed that *FOXO3* block-A haplotype 2 “GAGC” and haplotype 4 “AAAT” carriers had a higher risk of stroke (144). Mice subjected to transient middle cerebral artery occlusion (MCAO) developed severe cerebral infarction and long-term neurological deficit. FOXO3 overexpression was previously described in the cerebral cortical neurons of MCAO mice. Downregulation of miR-9 and miR-122 promoted neuronal death by up-regulating FOXO3 expression in the brain of MCAO mice (145, 146). Moreover, AMPK/FOXO3 and PTEN-Akt-FOXO3 pathways have been implicated in the regulation of neuronal apoptosis in response to hypoxia-ischemia during the developmental stages of rat brain (67, 147). In addition, the ischemia-reperfusion injury resulted in activation of FOXO3. Activated FOXO3 promotes autophagy, thereby reducing the injury caused by cerebral ischemia-reperfusion (148).

### The Role of FOXO3 in Primary Hypertension

Clinical studies have shown that vascular aging is a predictor and risk factor for hypertension. Patients with hypertension, regardless of whether their blood pressure is normal or not, are at an increased risk of developing cardiovascular events. Researchers have found that patients receiving antihypertensive drugs still have a 50% residual risk of cardiovascular death (149). The longevity-related *FOXO3* polymorphisms may be associated with lower blood pressure (BP) in Japanese women with hypertension (150).

### FOXO3 as a Promising Therapeutic Target in Aging-Related Vascular Diseases

FOXO3 is an ideal target for a variety of aging-related diseases, including cancer, degenerative diseases, and vascular aging. As previously described, FOXO3 is a good biomarker for cancer initiation, progression, and drug efficacy, and resistance. FOXO3 reactivation may be an efficient antitumor strategy. Furthermore, conditional deletion of *FOXO1/3/4* in mice triggered IVD degeneration, and therapeutic activation of FOXO could resist IVD degeneration by promoting stress resistance and autophagy (29). FOXO3 has a well-established function in the occurrence and pathogenesis of vascular aging-related diseases. The AMPK-FOXO3-Trx axis, which has been demonstrated to be a critical defensive mechanism against excessive generation of ROS induced by metabolic stress, may be a promising target in treating CVDs in metabolic syndrome (151). Akt inhibition activates FOXO3, which is also a good way to delay vascular aging (152). UCN-01, a drug currently used in clinical trials against cancer, inhibits Akt phosphorylation resulting in FOXO3 reactivation (153). UCN-01 was shown to be capable of reversing bleomycin- induced lung fibrosis *in vivo* by activating FOXO3 (153). Curcumin, a polyphenol, enhances FOXO3 function by inhibiting its phosphorylation, resulting in a two-fold increase in target gene expression (154). Further evidence confirmed that curcumin protects inflammatory cells in the vascular system against lipid and oxidant-induced damage by increasing FOXO3 activity, so lowering the risk for aging-related CVD (154). Additionally, human VECs, VSMCs, and MSCs expressing a constitutively active version of FOXO3 exhibited enhanced self-renewal capacity, greater regenerative capacity under ischemia conditions, and increased resistance to oxidative injury (155). The evidence presented above suggests that pharmacological reconstitution of FOXO3 may be a novel treatment option for aging-related vascular diseases.

Pharmaceutical regulation of the FOXO3 signaling pathway is a promising strategy to promote healthy longevity. It was found that FOXO3 longevity genotype mitigated the increased mortality risk in men with a cardiometabolic disease (CMD). Moreover, there was no association of *FOXO3* longevity genotype with lifespan in men without a CMD (17). Therefore, CMD patients without the *FOXO3* longevity genotype may benefit most from intervention targeting FOXO3. However, FOXO3 may not be a easily druggable target, because its activity is mediated by a complex network of interactions with other DNA, RNA, and proteins. Direct regulation of gene expression in a simple manner may not achieve the expected effect and cause redundant functions. FOXO3 activity is finely regulated by PTM modulators, which is a more feasible and acceptable therapy. Researchers have explored a number of agents identified as modulators of FOXO3 activity, including those that target nuclear export and import, drugs that target upstream regulatory targets, drugs that target FOXO3 protein interactions, and those that target DNA binding (156). FOXO3 exhibits variable affinity for target genes under different conditions (157). Consequently, the development of FOXO3-specific therapy based on multiple statuses is expected to improve efficacy and reduce the off-target effects. To improve the development of FOXO3-based treatment options, it is necessary to conduct additional studies on the regulatory networks, including upstream regulatory molecules and downstream pathways of FOXO3.

## Conclusion

Healthy aging is critical for addressing the increasing severity of global population aging. The unique role of FOXO3 in the vasculature provides promising avenues for therapeutics against aging-related vascular diseases. PTMs that regulate FOXO3 activity may be potential therapeutic targets. It is expected that research into strategies for delaying the occurrence and development of vascular aging by targeting the FOXO3 will uncover novel perspectives for the development of new drugs. Despite advances in our understanding of FOXO3's function in retarding vascular senescence, the particular processes remain poorly known, and other issues remain unresolved. For instance, FOXO3 activation promotes VSMCs apoptosis, which may result in atherosclerotic plaque instability and rupture, causing myocardial infarction, and cerebral infarction. In some cases, FOXO3 promotes ECM degradation which may accelerate the progression of atherosclerosis. While therapies targeting FOXO3 seem appealing, we need to understand all the details to maximize its effectiveness. Despite these challenges, in-depth research into FOXO3 functions may pave the way for future therapeutic approaches.

## Author Contributions

YZ collected the literature and wrote the manuscript. Y-SL conceived the idea and had been involved in manuscript conception and drafting. YZ and Y-SL read and approved the final manuscript. All authors contributed to the article and approved the submitted version.

## Funding

This work was supported by the National Natural Science Foundation of China (No. 82071593); the Fundamental Research Funds for the Central Universities of Central South University (No. 2021zzts0359); and Hunan Provincial Innovation Foundation for Postgraduate (No. CX20210128).

## Conflict of Interest

The authors declare that the research was conducted in the absence of any commercial or financial relationships that could be construed as a potential conflict of interest.

## Publisher's Note

All claims expressed in this article are solely those of the authors and do not necessarily represent those of their affiliated organizations, or those of the publisher, the editors and the reviewers. Any product that may be evaluated in this article, or claim that may be made by its manufacturer, is not guaranteed or endorsed by the publisher.
